# Identification of human African Trypanosomiasis foci using school-going children in post-conflict era in Nwoya District, Northern Uganda: A cross-sectional study

**DOI:** 10.12688/aasopenres.12851.1

**Published:** 2018-04-18

**Authors:** Kenneth Luryama Moi, James Henry Obol, Denis Anywar Arony

**Affiliations:** 1 Department of Medical Microbiology & Immunology, Faculty of Medicine, Gulu University, Gulu, Uganda; 2 Department of Public Health, Faculty of Medicine, Gulu University, Gulu, Uganda; 3 Department of Medical Biochemistry, Faculty of Medicine, Gulu University, Gulu, Uganda

**Keywords:** HAT, CATT/T. b. gambiense, Northern Uganda, Nwoya District, LAMP

## Abstract

**Background: **Human African Trypanosomiasis (HAT) is fatal if untreated; the drugs to treat it are toxic making its management difficult and diagnosis complex. Nwoya district has a long history of sleeping-sickness dating back to pre-colonial times. The civil war of 1986-2008 displaced many who upon return complained of cattle and dogs dying of unknown causes alongside increased tsetse flies infestation hence, the needs for the study.

**Methods: **We enrolled local 3,040 pupils and recorded their social-demographic characteristics and access to different domesticated animals/fowls in their homes. Screening for HAT using the card agglutination test for trypanosomiasis (CATT) was performed; positive individuals had their titres determined, followed by microscopy and loop mediated isothermal amplification analysis (LAMP). R was used for analysis where associations were sought between dependent and independent variables. Any factor with P-value <0.05 was taken as statistically significant.

**Results: **HAT serological prevalence of 1.2% (95% CI 0.8-1.6) was obtained, 58.3% being boys while 41.7% were girls with titres ranging from 1:2 - 1:16. Two schools alone, constituted 47% of the CATT positive cases.

Pupils who came from homes with dogs were more likely to be CATT/ *Trypanosoma brucei gambiense* positive; (adjusted odds ratio = 3.12, 95% CI 1.41-6.99 & p=0.005).

**Conclusions: **Though no parasites were detected, with prevalence of CATT positive at 1.2%, active surveillance in the district is still recommended. CATT positive cases needs follow-ups were immune trypanolysis test done to ascertain their exposure.

## Introduction

Human African Trypanosomiasis (HAT) commonly known as sleeping sickness is still active in over 30 sub-Saharan African countries ^1^, and found within the regions between latitude 14 ^0^ North and 20 ^0^ South in more than 250 active recognized different foci ^2^. If left untreated, it may lead to death ^3– 7^. The etiologic agents are; *Trypanosoma brucei rhodesiense,* the East African type that runs an acute course, and *T. b. gambiense,* the West African type that runs a chronic course ^4, 8^. The two species, unfortunately are morphologically undistinguishable, with characteristically different epidemiological features and drugs of treatment ^9, 10^. Up to 70 million people are at risk of this debilitating and fatal disease with 30,000 individuals estimated to be infected ^11^. Since 2010, *T. b. gambiense* was still endemic in at least 24 countries in Africa, and accounted for over 95% of HAT reported cases ^2, 7, 12^.

Uganda is the only country that harbours both species ^1, 13^, with *T. b. rhodesiense* having afflicted the South, through Eastern into mid-northern Uganda, while *T. b. gambiense* is endemic in North-West Nile districts and part of the Amuru district ^12^.

In Northern Uganda, HAT was the main cause of relocations, a policy of compulsory evacuation of the natives from the affected areas ^4, 14^, an action that remained in force until the early 1950’s ^15^. Large swaths of land, especially in Anaka and Purongo sub-counties, had to be bush cleared in an attempt to rid the vectors of suitable habitation (The Monitor Newspaper of 22 ^nd ^Nov. 1999). Tsetse Control Camps were permanently maintained in several places that bordered the Murchison National Game Park and East Madi game reserve up to the period just before the northern insurgency in 1986.

Over two decades of civil war (1986–2008) led to mass displacement of people leading to total breakdown of social and physical infrastructures ^16^. This same region is reported to be a potential site for a possible overlap of the two pathogenic *brucei* subspecies estimated to be within 160 km apart, and yet the so called frontier that divides the two seems to be only imaginary ^1, 2, 17^.

Our study was therefore designed to use primary school-going children to ascertain the prevalence of HAT, identify and map the current possible HAT foci, and to determine if there is any association among card agglutination test for trypanosomiasis (CATT) positive individuals and domestic animals/fowls kept at homes.

## Methods

### Study area

Nwoya district is located in mid-northern Uganda and lies within 02 ^0^38N, 32 ^0^ 00E covering 4,736.2 km ^2^ (1,736.2 sq miles) with a population of 54,000 people. Subsistence agriculture and livestock husbandry is the main economic activities besides tourism however; currently, there is steady increase in commercial agriculture according to Uganda Bureaus of Statistics, 2011.

The district is made up of 4 sub-counties (Koch Goma, Alero, Anaka and Purongo) in addition to Nwoya town council. Koch Goma and Purungo sub-counties form part of Murchison National Game Park; and thus are heavily infested with tsetse flies which act as vectors for sleeping sickness. In 2013, there were 44 Government-aided primary schools in the district with 39,632 pupils; 15,428 being boys while 14,436 were girls according to records obtained from district education office.

### Study design

Our study was a cross-sectional survey; questionnaire was used to collect socio-demographic characteristics and risk factors while laboratory form was used for registering blood sample. The study was conducted in schools in sub-counties that form parts of the Murchison National Game Park and those closer to River Nile, were eligible for sampling. In total we purposively selected 19 Government-aided and 2 community schools since they are closer to the game park or river Nile which are foci for tsetse flies. In each school, we recruited pupils using consecutive sampling methods in each class. Pupils were drawn from primary three to primary seven, except for Gony-Cogo community school that had the whole school enrolled due to their small numbers and the children generally being more mature with one only who was 4 years old.

### Data collection

***Socio-demographic characteristics*.** Data on socio-demographic characteristics of each pupil; past history (e.g. places where they could have lived other than their homes), and the different animals/fowls kept at home were recorded. A Global Positioning System (GPS) was used to record locations of salient features; schools, sub-county headquarters, health units, district headquarter and homes of pupils who were found to be CATT/ *T.b. gambiense* positive for easy follow-ups in the future.

***Blood collection and preparation*.** At each selected schools, ethical procedures were observed e.g. explaining the purpose of the study and why they needed to participate although they would experience some slight pain during sample collection. After obtaining their assent, they were then enrolled by giving them identification numbers before going through the questionnaires to capture demographic information as well as animals/fowls kept at home. Finally, 2–3 ml of whole blood was collected aseptically following vein puncture in the *cubital fossa* into sterile plasma tubes 4.0 ml (BD, Franklin Lakes, NJ, USA) spray-coated with 60 USP (Units of Sodium Heparin) as an anticoagulant ^18^. The blood was gently homogenized with the anticoagulant, 3% Phosphate Saline Glucose (PSG) was added to keep the trypanosomes active for a prolonged period. Samples were kept in cold boxes at temperature not exceeding 20 ^0^C to avoid exposure to heat and direct sunlight.

***CATT test and microscopy*.** Screening was performed using CATT/ *T. b. gambiense* in accordance to the manufacturer’s (ITM, Antwerp, Belgium) manual by diluting blood 1:2 in CATT buffer. Briefly, a drop of whole blood was mixed with a corresponding amount of the reagent and rotated onto a flat orbital rotator for 5 minutes at 60 rpm. Both positive and negative controls were set along test samples and results read as positive, if there were visible agglutinations with the naked eye ^19, 20^. Titres were obtained by making twofold dilutions of 1:4, 1:8, 1:16 and 1:32 plasma in CATT buffer. 25 µl freshly reconstituted CATT/ *T. b. gambiense* reagent (Institute of Tropical Medicine, Antwerp, Belgium) was added to each dilution, mixed and rocked for 5 minutes at 60 rpm. Titre was read as the highest dilution where visible agglutination was observed.

Wet preparations were made, mounted with cover slips 24 × 32 mm and examined using Olympus CX21 microscope under x20 and x40 magnifications and the results recorded. Thick blood smears of CATT positive samples were made, air dried, labeled and packaged into slide folders for eventual staining with *Giemsa* stain (SIGMA-ALDRICH ^®^, Catalog No. GS) for laboratory examinations under oil immersion (x100).

***Sample preparations & detection of repetitive insertion mobile element (RIME) using loop mediated isothermal amplification (LAMP)*.** Homogenized whole blood from plasma tubes were sucked in heparinized capillary tubes sealed at one end with plasticine and spun at 800 x g (M24 Hematocrit centrifuge, LW Scientific) for 10 minutes to separate the different blood constituents. Buffy coats are located at the interface of packed cells on the lower end and serum at the top end. Using diamond pencil the tube is cut just above the packed cells and the Buffy coat was applied carefully onto labeled FTA ^®^ Classic Cards (Lot No. 5114552C, Whatman International Ltd, Maidstone, UK) beginning from the centre moving outwards within the circle, air dried, packaged in self-sealing plastic bags containing desiccants, and stored in a lockable cupboard/drawer for LAMP analysis.

Loopamp ^TM^ *Trypanosoma brucei* detection kit *version3.11* was used to carry out the analysis by following the most recently revised standard protocol ^21, 22^. Test sample was positive if florescence was present indicating the availability of trypanosome DNA and negative if there was no fluorescence ^21– 23^. Both positive and negative controls were checked to confirm the validity of the test before results were read and recorded.

### Data management and analysis

Data were entered in *Microsoft Office Excel 2007*, exported to R *version 3.2.3* converted to comma delimitated (.csv) file, cleaned, edited and exported to STATA version 11 for analysis. Continuous variables were summarized using mean and categorical data were summarized in terms of frequencies and percentages. Univariate analysis was performed for both dependent and independent variables. Prevalence of HAT was obtained by dividing those who were CATT positive with the total number of pupils screened. Bivariate logistic regression was performed to determined association between the independent variables and presence of HAT. We reported Odds Ratio, 95% CI and P-value. Multivariate logistic regression analysis was used to assess for association between presence of HAT and the independent predictors. We run the stepwise logistic regression while adjusting for other independent variables in the model. We also adjusted for clustering of data around schools since the study was a survey. We calculated the adjusted Odds ratio, 95% confidence interval and P-value. Any variable with P-value ≤0.05 was taken as a significant predictor of HAT presence.

### Ethical clearance

Ethical clearance (Ref. No. GU/IRC/01/11/11) was sought from Institutional Review Board of Gulu University. Study approval was granted by the Uganda National Council for Science and Technology (UNCST). The school management (Head teacher) was briefed about the study and the pupils were briefed about the study by the investigators. After thorough explanation in Acholi, the local language, the purpose of the study and the procedure involved, the pupils were given an informed consent form to take to their parent and return with it the following day when reporting to school before the study was conducted. The pupils who accepted to participate in the study signed an assent form. Pupils whose parents did not provide consent were excluded from the study and pupils who expressed fear of being pricked with needle even if their parent had consented and had first assented to participate in the study were excluded from the study, because that was a sign of withdrawal of assent. Children who were found to be CATT positive were followed home for a brief of their parents about the results and offer guidance where to seek treatment.

## Results

A total of 3,040 pupils were enrolled for participation; 49.4% (n=1,501) were boys and 50.6% (n=1,539) were girls. The prevalence of CATT/ *T. b. gambiense* positive was 1.2% (n=36), (95% CI 0.8 – 1.6%); 58.3% (n=21) were boys while 41.7% (n=15) were girls. All the 36 pupils had neither parasites detected in their blood by microscopy, nor did they show swollen lymph nodes on palpation. Table 1, below summarizes the results for schools surveyed.

**Table 1.** Primary schools surveyed in Nwoya district along with their CATT results and titres.

Sub-County	Primary Schools accessed	No. Screened	CATT +ve	TITRES			
				1/2	1/4	1/8	1/16
Koch Goma	Wlilacic	224	3	1	0	1	1
	Goro	96	0	0	0	0	0
	Koch Goma	211	1	0	1	0	0
	Koch Lii Pakiya	125	0	0	0	0	0
	Koch Lii	197	0	0	0	0	0
	Koch Lila	169	4	2	0	0	2
	Lutuk Community	128	8	2	3	2	1
	Gony Cogo Comm	62	1	0	1	0	0
Purongo	Got Apwoyo P/S	193	0	0	0	0	0
	Wii Anaka P/S	146	5	3	1	1	0
	Purongo P/S	186	1	0	0	0	1
	Paraa P/S	212	9	1	7	1	0
	Olwiyo P/S	128	1	0	1	0	0
	Purongo Hills P/S	219	1	0	0	0	1
	Aparanga P/S	149	0	0	0	0	0
Anaka	Agung P/S	142	1	0	0	1	0
	St Kizito Bidati P/S	159	1	0	1	0	0
Alero	Lulyango P/S	153	0	0	0	0	0
Lungulu P/S		144	0	0	0	0	0
**4**	**19**	**3043**	**36**	**9**	**15**	**6**	**6**

Paraa P/S in Purongo and Lutuk Community School in Koch Goma sub-counties had the highest number of pupils who tested CATT positive with 9 and 8 respectively. Those with titre ¼ (moderate reactions) were 15 while those with strong reactions 1/8 and 1/16 were 12.

School enrolment is at 50–50 for girls and boys although those involved in the study where mainly in the age group 13–18 years (63%). Fowls/animals that are mostly kept at home in the district are chicken, goats, and dogs with pigs and cattle are steadily on the increase over the years ( Table 2).

**Table 2.** Socio-demographic characteristics of the pupils that participated in the study and the animals/fowls kept at home.

Variables	Frequency	Percentage
**Sex**		
Male	1,501	49
Female	1,539	51
**Age groups**		
4 – 12 Years	1,120	37
13 – 18 Years	1,921	63
**Presence of dogs at home**		
Yes	1,742	57
No	1,299	43
**Pr** ***esence of chicken at home***		
Yes	2,811	92
No	230	8
**Presence of ducks at home**		
Yes	465	15
No	2,576	85
**Presence of turkeys at home**		
*Yes*	92	3
No	2,949	97
**Presence of cattle at home**		
Yes	925	30
No	2,116	70
**Presence of goats at home**		
Yes	2,482	82
No	559	18
**Presence of sheep at home**		
Yes	602	20
No	2,439	80
**Presence of pigs at home**		
Yes	771	25
No	2,270	75

**Figure 1.**
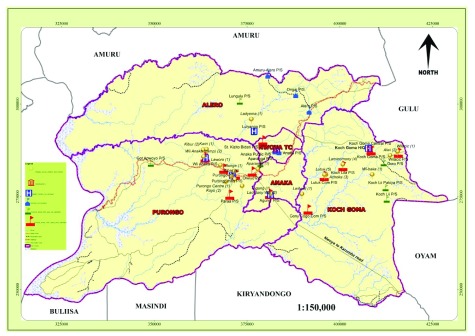
Map of Nwoya district showing sub-counties boundaries, schools accessed, and physical features such as rivers, roads, health units, and GPS locations of homes of CATT positive cases.

With the use of GPS; villages, schools, sub-counties, district headquarter, hospital/health centres and salient features such as district and sub-county boundaries, road networks and streams/rivers are located on the district map for ease of follow ups especially the CATT positive cases even by someone who has not been involved in the study ( Table 3; Figure 1).

**Table 3.** Crude Odds Ratio and Adjusted Odds Ratio for the predictor of being CATT positive.

Variables	c OR	95% CI	P-value	a OR	95% CI	P-value
Chicken	0.40	0.17 - 0.98	0.044	0.47	0.18 - 1.24	0.128
Ducks	0.69	0.24 - 1.96	0.486	0.64	0.22 - 1.90	0.423
Turkeys	1.91	0.45 - 8.05	0.381	2.59	0.56 - 11.87	0.221
Cattle	0.88	0.42 - 1.83	0.729	0.95	0.43 - 2.07	0.902
Goats	0.51	0.25 - 1.04	0.063	0.49	0.22 - 1.12	0.089
Sheep	0.98	0.43 - 2.24	0.958	1.09	0.46 - 2.62	0.843
Pigs	0.59	0.24 - 1.41	0.234	0.57	0.22 - 1.43	0.229
**Dogs**	**2.26**	**1.06 - 4.81**	**0.035**	**3.12**	**1.41 - 6.94**	**0.005**

**Figure 2.**
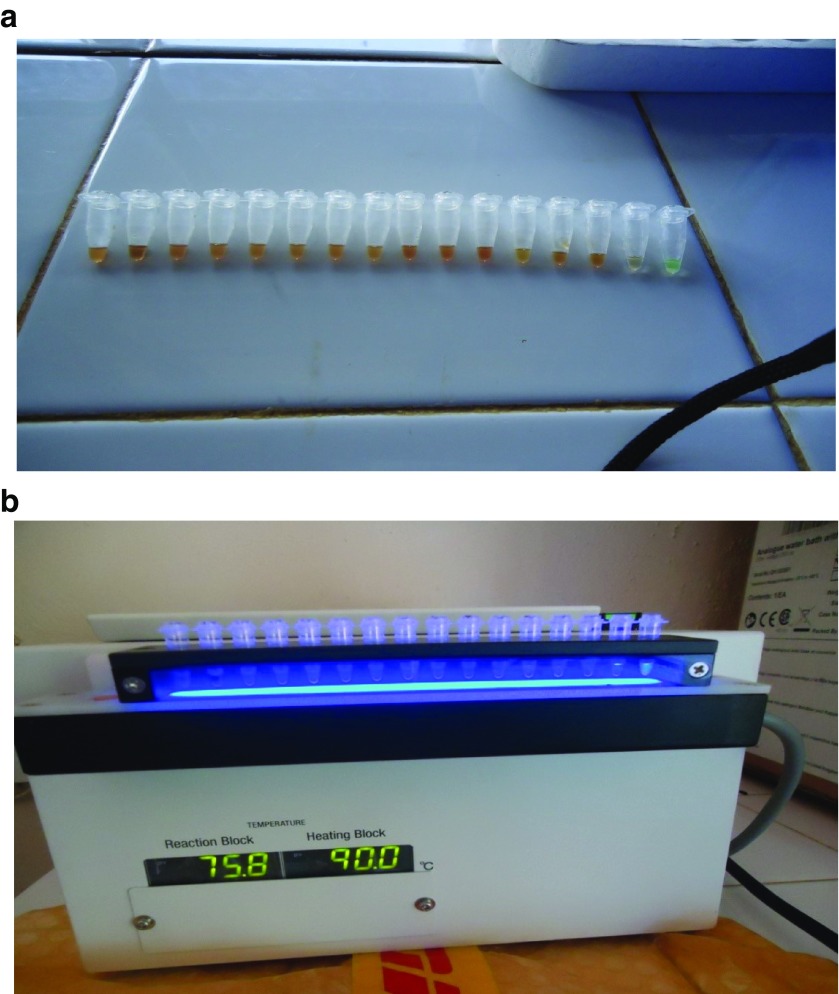
( **a**) Prepared tests samples set along with both negative and positive controls in reaction tubes ready for reading before being inserted in visualization chamber. Tubes labeled 1 to 14 (from left to right) are test samples; tube 15 is a negative control (colorless) and tube 16 is a positive control (Light green). ( **b**) Picture showing test samples in a fluorescence unit of the LAMP incubator. Tubes 1 to 15 showing no fluorescence in the test and negative control fluids, whereas there was intense fluorescence in tube 16 (positive control) as seen to the naked eye.

Of the 36 CATT/ *T. b. gambiense* positive samples, 34 were subjected to LAMP and all of them were found to be negative. The results were as shown in Figure 2a and 2b.

## Discussion

A study conducted in Taraba state, Nigeria screened n=400 using CATT/ *T. b. gambiense* obtained a prevalence of 1.8% (7/400). Males tested more positive than their female counterparts ^23^ and the difference in the number of males and females who were CATT positive was statistically significant, p=0.05. Although this agrees with our study; males (21/36) being more CATT positive than females (15/36), the difference in the sexes was not statistically significance. Due to a small sample size of Taraba study, this could have influenced the prevalence causing a significant difference between the sexes in terms of infectivity. HAT is a disease that is related to behavioural risk factors through complex interactions; environmental and behavioural risk factors, vector and the human host ^24^.

Considering the fact that our subjects were school-going children, they were engaged in similar activities in disregard to their sexes and this could have been a key factor of having no statistical difference in the way the two sexes were exposed. In villages where girls go out in search of firewood in the park or forests, as seen in Laworo and Wii Anaka in Purongo sub-county, they got more exposed than the boys, whereas in areas where boys herd animals, go digging or playing in the fields, they were more exposed than girls, as observed in Alwi and Laminomony villages in Koch Goma sub-county. In some cases, pupils who travelled long distances leave schools nearby; Paraa Primary School (P/S)coming from Purongo trading centre, St Kizito Bidati P/S from Ladyema in Alero sub-county, Olwiyo P/S from Aparanga and Koch Goma P/S from Wi-Baka. These pupils were likely to have been exposed along the way as they travelled to school; indicated by the fact pupils who attended schools nearby were CATT negative. 

The validity of CATT results has been a subject of controversies in the wake of low endemicity since it’s known to work well in endemic areas with prevalence above 5% ^25^. In our study however, the distribution of pupils who were CATT positive had a very striking pattern; in most cases, where there were several positive cases arising from a particular school, the pupils tended to come from the same villages as was observed in the villages of Laminomony, Wii Anaka area, Lutuk, Kibar, Koyo and Alwi. In one scenario, 2 cases from Lutuk community school were siblings from the same homestead in Bargunya village. Lutuk had a tsetse control camp in the 1980’s due to HAT outbreak then, these results could indicate that it is on the verge of HAT re-emergence once again. While Wii Anaka and Paraa are settlements close to Murchison National Game Park; a place known to be infested with tsetse flies. Like Lutuk, Wii Anaka also had a nearby tsetse control camp at Got Apwoyo that now lies in ruins. Our findings therefore, reconfirms that most of the CATT positive results were certainly not due to errors or cross-reactions as other previous authors have suggested ^13^, it’s likely that these children could have been exposed, and either the parasites failed to be maintained or got neutralized by their strong immunity due to their long period of stay in this HAT endemic region ^26^. It’s also likely that over time *T. b. gambiense* could have under gone a lot of changes while in the human host; a study conducted in northern Uganda had found that 75% of those who were found to be CATT positive where parasitologically positive with careful wet preparations and thick blood smear microscopy ^27^. Currently, numerous studies are reporting lots of aparasitaemic cases with gross variations in parasitaemia across foci ^24, 28^. Humans, like animals, are believed to possess trypanotolerance that protect them from the disease ^29^. 

Cut-off titres at some points were used to define disease cases; 1:4 required parasitological confirmation whereas 1:16 was regarded as indication of infection that required treatment even without parasites being seen ^13^. In our study the following CATT positive cases were detected; 1:2 (n=9), 1:4 (n=15), 1:8 (n=6), and 1:16 (n=6) (see Table 1). Our findings cast doubts on reliability using CATT titres, as high as 1:16 did not reveal presence of parasites or their DNA. In a related study that was done in 2012, a case from Onigo village, Miniki sub-county in Adjumani district that was CATT positive with a titre of 1:4 who was aparasitaemic at the time, turned out to be a stage 2 case after trypanosomes were recovered from his CSF within 2 weeks from the time CATT screening was performed [unpublished study, Luryama Moi K, Anywar D and Madra P].

Though it has now been proven that animals play a yet unclear role in the maintenance of *T. b. gambiense* even when they have been eliminated from the human population, explaining in parts the reason behind re-emergence of HAT ^30^. In a study conducted in West Africa, out of 397 domestic animals sampled, 64% were CATT positive for *T. b. gambiense* and when PCR analysis was done, 15.4% of sheep, 11.6% of pigs, 3.5% of goats and a low number of dogs where confirmed as infected with HAT ^31^. Meanwhile a study carried out in Cameroon did not find any *T. b. gambiense* infection in dogs; this could have been due to a small number of dogs sampled ^32^. In our study however, we did not sample the animals but instead tried looking at possible associations of those pupils who were CATT positive and the domesticated animals. After analysis for possible associations we found that those who had dogs were more than 3 times at increased risk of being CATT positive; Adjusted OR 3.12, (95% CI 1.41-6.99 and p = 0.005). This relationship could be explained in two ways; dogs could be acting as a reservoir of *T. b. gambiense*, a threat to re-introduction into human population. The other explanation could be due to the fact that dogs are social animals that are routinely used for hunting and herding animals in this region, and it could simply mean that those with dogs spend more time outside and therefore at higher risk of exposure than those without dogs.

All our CATT positives turned out to be negative by LAMP even those with high titres of 1:16. Studies in one of the sleeping sickness treatment centre at Omugo Health Centre (level IV) located in Arua district, north-western Uganda where SD BIOLINE HAT ^TM^ (Alere Inc., Waltham, MA, USA) is currently used to test for *T. b. gambiense* VSG *LiTat* 1.3 and 1.5 antibodies found that; out of 72 SD BIOLINE positive, only 2 were positive RIME LAMP [unpublished study, Luryama Moi K and Louga A]. As suggested by Mitashi *et al.* (2013), we also recommend that LAMP may still require more evaluation studies before it’s adopted as a gold standard in the diagnosis of *T. b. gambiense*. 

### Study limitations

This was a one off survey and therefore we cannot account for information on pupils who refuse to participate or were absent from school nor children who have dropout of schools. However, our sample was large enough to be representative of the general pupils’ population in the schools in the sub-counties which form foci for tsetse fly.

The screening test used only targeted *T. b. gambiense* and as such making only 36 samples to qualify for testing using LAMP that detects both *T. b. gambiense* and *T. b. rhodesiense*. However, the information is good enough for baseline data upon which future research can build on.

### Recommendations/conclusion

Pupils who were CATT/ *T. b. gambiense* positive need a follow-up study for repeat CATT test, as well as performance of an Immune trypanolysis test to establish their infectivity status as the latter test seems to be more accurate. There is need to for active screening of populations using superior screening tests that combines both, *T. b. gambiense* and T *. b. rhodesiense* for regions that faces the threats of merger. Health workers at lower health facilities need sensitization and skill development in identifying cases with HAT. In conclusion, we confirm that the use of school-going children offers the most efficient means in the identification of HAT foci in places that accessibility is difficult or participation from the community is low.

## Data availability

Data underlying the study are available on OSF: http://doi.org/10.17605/OSF.IO/DM7FT ^33^

Data are available under the terms of the Creative Commons Zero "No rights reserved" data waiver (CC0 1.0 Public domain dedication).
