# Supplementary material for: Identification of human African Trypanosomiasis foci using school-going children in post-conflict era in Nwoya District, Northern Uganda: A cross-sectional study
Source: AAS Open Res. 2018 Apr 18;1:8. [Version 1] doi: 10.12688/aasopenres.12851.1 (PMC7194148; doi:10.12688/aasopenres.12851.1)
Supplement: Peer review history

# Peer review: Reviewer response for version 1

**DOI:** 10.21956/aasopenres.13917.r26327

**Published:** 2018-05-08

**Authors:** Enock Matovu

**Affiliations:** 1 Department of Biotechnology and Diagnostic Sciences, College of Veterinary medicine, Animal Resources and Biosecurity, Makerere University, Kampala, Uganda

The manuscript describes a school-based approach to investigate HAT. This approach (unlike in the case of diseases such as schistosomiasis) is not normally taken in HAT, perhaps because most cases affected are among the middle age-group as a consequence of longer-term exposure via occupational activities that bring them into contact with the Tsetse fly. Nevertheless, the authors have shown that taking a school-based approach, one is able to zero in on microfoci where transmission might be taking place.

 In the background part of the abstract, it is indicated that cattle and dogs were dying of undiagnosed disease, in presence of heavy tsetse infestation. It would have been interesting to do a pilot sampling of these to show any evidence for trypanosomes as a basis to suspect possible presence of HAT. But dogs are known to be susceptible to *T. brucei* group and typically die of symptoms similar to those of late stage HAT, corneal opacity is also a common manifestation of Trypanosomiasis in dogs.

 CATT is an antibody test, known to cross react with other parasite antigens. Thus false positives are expected, even when executed outside HAT endemic foci. However, its specificity inside HAT endemic areas is considerable high, making it a useful test to screen for potential HAT cases. The authors should make it clear that they investigated sero-prevalence rather than HAT prevalence since a CATT positive reaction does not exclusively point to HAT presence. There is also need to use generally accepted terminologies e.g. Their CATT 1/2 might be referring to CATT whole blood. Normally in screening we talk about CATT whole blood, CATT ¼, 1/8, and 1/16; 1:4 is not the same as 1/4. Also clarify that the wet smears were read under x20 and x40 objectives; the magnifications were x200 and 400 respectively. Noteworthy is that being an area at the periphery of a T. b. gambiense endemic districts, more sensitive parasitology such as the heamatocrit centrifugation technique (HCT) would have been more appropriate, given the characteristically lower parasiteamia of that subspecies in humans.

 For LAMP, the authors give no details of how they processed template before running the test, so it is not clear if it was performed with the best possible template. LAMP is a good molecular test but can not be considered a gold standard. The HAT case definition set by the WHO remains demonstration of the parasite itself in blood, lymph node aspirates, or cerebrospinal fluids; these comprise the so-called “composite reference standard”. The authors could also have considered PCR alongside, to show if any signals indicative of T. brucei would have been obtained.

 In the discussion, please refer to seropositive individuals as suspects rather than cases.

 The major arguments in the discussion regarding trypanotolerance would hold water, however in absence of any evidence for human infective trypanosomes circulating in humans, animal reservoirs or vectors in Nwoya, they remain very highly speculative. The only pointer to HAT is the CATT seropositivity whose results alone remain far from conclusive (it is a screening test that must be follwed by confirmatory parasitology); more techniques need to be deployed in the area in order to make more valid conclusions.

 Other minor comments: use either “North west Uganda” or “West-Nile” districts. The words “were” and “where” have been interchangeably used, please rectify. Under recommendations, TL would help establish “infection”, not “infectivity” status.

I confirm that I have read this submission and believe that I have an appropriate level of expertise to confirm that it is of an acceptable scientific standard, however I have significant reservations, as outlined above.

# Peer review: Reviewer response for version 1

**DOI:** 10.21956/aasopenres.13917.r26363

**Published:** 2018-05-08

**Authors:** José R. Franco

**Affiliations:** 1 Control of Neglected Tropical Diseases, Innovative and Intensified Disease Management, World Health Organization, Geneva, Switzerland

There are important conceptual misunderstandings in the manuscript reviewed that can give a wrong impression about the epidemiological situation of HAT in the area studied.

1. The CATT is a serological test used for screening and therefore with a specificity limited and well described cross reactions (false positives), as for instance with transient infections by nonhuman trypanosomes ^1^. This could be the case in the studied area. In areas of very low or zero HAT prevalence, the predictive positive value of the test is very low and the presence of CATT seropositives does not give any indication about the presence of HAT. No trypanosome was observed in the serological suspects after parasitological test, and therefore with the data presented it is not possible to infer that there is a presence or risk of the disease in the area. To talk than about “HAT serological prevalence” is confusing and wrong (The authors define “Prevalence of HAT” as CATT positive individuals divided by screened population). The statement indicating that these CATT results are not due to errors or cross reactions is not adequately justified.
2. Nwoya district was affected in the beginning of 20 ^th^ century by gambiense sleeping sickness ^2^. In different outbreaks in the mid-20 ^th^ century, the distribution of cases did not reach the area of Nwoya (Jonan and Okoro districts) ^3^. At the end of the last century (from 80’s), an important outbreak of gambiense HAT was described in West Nile ^4^, but no cases of gambiense HAT have been diagnosed in the area of Nwoya since that time ^5^^,^^6^^,^^7^.
3. Sporadic cases of rhodesiense have been diagnosed in tourists visiting the neighbouring National Park of Murchinson Falls but these cases are due to *T. b. rhodesiense* (e.g. http://www.nicd.ac.za/assets/files/NICD-NHLS%20Communicable%20Disease%20Communiqu%C3%A9_DECEMBER%202014.pdf) and linked to the presence of *T.b rhodesiense* in wild animals. To investigate local populations with gambiense CATT would not have any interest to asses’ rhodesiense HAT infections, which could happen in the studied area. At the same time primary school children are not a group at high risk for rhodesiense HAT, usually linked with activities related to wild animals and people entering in the protected areas (rangers, poachers, tourist guides, herders, honey and firewood collectors,…). 
4. It is important to clearly distinguish between gambiense and rhodesiense HAT and also to underline that the presence of tsetse fly and even animal trypanosomiasis does not implies the presence of human African trypanosomiasis.
   
  1. The presence of animal trypanosomiasis in the area is well-known ^8^.
  2. The presence of *T.b gambiense *has been described in different animals but the epidemiological role that animals can play as reservoir for gambiense HAT is not clear ^9^.
5. There would be an ethical concern as if the authors consider the CATT seropositives as possible cases and no action has been taken to follow them up to confirm the presence or not of a disease that is considered as lethal.

 

 The authors confirm “that the use of school children offers the most efficient means in the identification of HAT foci in places that accessibility is difficult or participation from the community is low”. According to the existing epidemiological data, the district of Nwoya cannot be currently considered as a HAT focus. The data presented in the paper (some children presenting CATT positive reactions but no presence of trypanosomes after parasitological tests) does not allow considering the area as a focus with active transmission of HAT.

 The study tries to ascertain the prevalence of HAT using primary school children, and the conclusion should be that just based in some CATT positive results, it is not possible to conclude that there is transmission of HAT in the area.

 It is not clear if authors want to assess the situation of gambiense HAT or rhodesiense HAT, and it would be important to clarify it.

I confirm that I have read this submission and believe that I have an appropriate level of expertise to state that I do not consider it to be of an acceptable scientific standard, for reasons outlined above.

## References

[1] : Options for field diagnosis of human african trypanosomiasis. *Clin Microbiol Rev*.2005;18(1) : 10.1128/CMR.18.1.133-146.2005 133-46 10.1128/CMR.18.1.133-146.2005 15653823PMC544181

[2] : Studies on the epidemiology of sleeping sickness in East Africa. 5. Sleeping sickness in the Bunyoro District of Uganda. *Trans R Soc Trop Med Hyg*.1960;54:585-96 1377284910.1016/0035-9203(60)90036-5

[3] : The epidemiology of sleeping sickness in East Africa. *Transactions of the Royal Society of Tropical Medicine and Hygiene*.1962;56(4) : 10.1016/0035-9203(62)90055-X 316-338 10.1016/0035-9203(62)90055-X 14476265

[4] : Sleeping sickness in Uganda: revisiting current and historical distributions. *Afr Health Sci*.2006;6(4) : 10.5555/afhs.2006.6.4.223 223-31 10.5555/afhs.2006.6.4.223 17604511PMC1832067

[5] : La trypanosomiase à Trypanosoma brucei gambiense dans le foyer du Notd-Ouest de l'Ouganda: bilan de 5 années de lutte (1987-1991). Commentaire. *Bulletin de la Société de pathologie exotique*.1995;38-41 7787452

[6] : Final evaluation of the MSF-France trypanosomiasis control programme in West Nile, Uganda. *Paris: Epicentre*.2002;

[7] : The Atlas of human African trypanosomiasis: a contribution to global mapping of neglected tropical diseases. *Int J Health Geogr*.2010;9: 10.1186/1476-072X-9-57 57 10.1186/1476-072X-9-57 21040555PMC2988709

[8] : Heterogeneity in the prevalence and intensity of bovine trypanosomiasis in the districts of Amuru and Nwoya, Northern Uganda. *BMC Vet Res*.2015;11: 10.1186/s12917-015-0567-6 255 10.1186/s12917-015-0567-6 26449544PMC4599665

[9] : Do Cryptic Reservoirs Threaten Gambiense-Sleeping Sickness Elimination?. *Trends Parasitol*.2018;34(3) : 10.1016/j.pt.2017.11.008 197-207 10.1016/j.pt.2017.11.008 29396200PMC5840517

# Peer review: Reviewer response for version 1

**DOI:** 10.21956/aasopenres.13917.r26362

**Published:** 2018-04-30

**Authors:** Mike Brown

**Affiliations:** 1 London School of Hygiene & Tropical Medicine, London, UK

This is a useful surveillance study covering an important region, from which there have been few data published in the last 15 years. It addresses an important public health issue which is the potential role of domestic livestock in limiting the effectiveness of HAT elimination programmes. The methodology used was good, and the analyses and conclusions fair.

 I have the following comments covering sections that would need major revision.

1. The introduction, while providing some background on HAT in Uganda, is not sufficiently focused on the question of interest which is the role of domestic animals in HAT transmission. It needs to be shortened and re-focused on this topic.
2. There is little data here on current knowledge (or lack thereof) of active case finding studies in this area i.e. is the current study embedded within an existing surveillance programme or is there no current active case finding there, and what did prev data show? What does passive case finding data demonstrate to justify looking in this school-age population to answer the question around domestic livestock. Why schoolage children?
3. It does not follow, to the reader, why "near Murchison.....thus are heavily infected....". Please clarify
4. What was delay from sample collection to wet microscopy?
5. What was access to treatment? It would be unethical to embark on this screening strategy without positive participants being able to access treatment. A short sentence describing treatment provision in the area would be appropriate.
6. Paragraph 4 of results. It is unclear what this has to do with results. I do not understand the paragraph.
7. It is unclear what potential explanatory factors were analysed in univariate analyses, that were then included in multivariate model. Were there measures of socioeconomic status? Were all the different animals included in the model? if so why? i.e. what is the logic of adjusting for e.g. chickens in exploring relationship between dogs and CATT positivity? It looks like this has been done but am not sure it is appropriate. If you collected GPS data shouldn't you have adjust for e.g. distance from Murchison or from Nile?
8. In the discussion I think the authors need to convince more fully that CATT positivity meant current HAT infection. None of the positive subjects had any other tests to support the diagnosis, and a serological test is poor evidence in itself of infection, esp in a setting where people may be more exposed to other infections that might drive hypergammaglobulinaema e.g. malaria, HIV, etc. I think that the real story in this paper is (A) domestic livestock have been shown to harbour HAT and this may have a role in HAT transmission. (B) in an area where there has historically been a strong HAT public health problem and despite control programmes and treatment access this has not completely disappeared, domestic livestock could be a reason for ongoing infection (C) we looked for associations between potential HAT infection/exposure as measured by CATT and domestic animals (D) we found an association esp with dogs BUT (E) no patients were parasitaemic and so there are limitations on whether these were true cases and (F) most cases were found in wards closest to large tsetse populations  -  dogs/etc may not have been on the causal pathway but may reflect confounding by activities such as hunting, socioeconomic status, etc.
9. There is a publication in Vet Parasitol 2012 (Balyeidhusa et al) showing no PCR evidence of human tryps in animals in NW Uganda. This has not been referenced or discussed.
10. PCR is a tool increasingly used (see studies by Jammoneau et al for example) to detect true infection in aparasitaemic CATT positive patients. Authors might recommend this technique in their concluding remarks.

I confirm that I have read this submission and believe that I have an appropriate level of expertise to confirm that it is of an acceptable scientific standard, however I have significant reservations, as outlined above.

# Peer review: Reviewer response for version 1

**DOI:** 10.21956/aasopenres.13917.r26364

**Published:** 2018-04-30

**Authors:** Joel L. Bargul

**Affiliations:** 1 Department of Biochemistry, Jomo Kenyatta University of Agriculture and Technology, Nairobi, Kenya

**Abstract**

**Conclusions (under Abstract): Pg 1**

 “CATT positive cases needs follow-ups were immune trypanolysis test done to ascertain their exposure” This sentence is unclear, thus re-write to correct grammatical mistakes.

**Keywords:** **Pg 1**

 CATT/T. b. gambiense = Follow rules when writing a scientific name. The genus and species names are always italicized; *T. b. gambiense*

**Introduction: Pg 3**

 1 ^st^ paragraph, 3 ^rd^ sentence- the word ‘runs’ is repeated two times in the same sentence. In the 4 ^th^ sentence, substitute *‘undistinguishable’* with the more commonly used *‘indistinguishable’*

 3 ^rd^ paragraph, 1 ^st^ sentence; In Northern Uganda… = In northern Uganda…

**Methods: Pg 3**

 Study area

 Re-write the second sentence in the 1 ^st^ paragraph;

*Subsistence agriculture and livestock husbandry is the main economic activities besides tourism however; currently, there is steady increase in commercial agriculture according to Uganda Bureaus of Statistics, 2011.*

**Study design: Pg 3**

 1 ^st^ sentence on 1 ^st^ paragraph;

*Rephrase the following sentence*

 Our study was a cross-sectional survey; [revise usage of semi-colon] questionnaire was used to collect socio-demographic characteristics [data] and risk factors[,] while laboratory form was used for registering blood sample [laboratory forms were used to record the details of collected blood samples].

 In the 2 ^nd^ & 3 ^rd^ sentences, include punctuation marks (e.g. commas) where appropriate [‘In total [insert a comma] we…’], in another case, a comma was wrongly inserted into the sentence (see ‘… and those closer to River Nile, [delete comma] were eligible…’).

 Last sentence under study design;

 Pupils were drawn from primary three to primary seven, except for Gony-Cogo community school that had the whole school enrolled due to their small numbers and the children generally being more mature with one only who was 4 years old.

 Question;

 How did you define maturity? What age bracket is mature? This statement on maturity is subjective.

**Data collection: Pg 3**

*Socio-demographic characteristics.[:]* Data on socio-demographic characteristics of [delete to avoid repetition]; past history (e.g. places where they could have lived other than their homes [ *insert*: within the study sites]), and the different [domestic] animals/fowls kept at home were recorded [ *insert:* for each pupil].

*Blood collection and preparation*

 Rephrase the following statement;

 “After obtaining their assent, they were then enrolled by giving [= assigning] them [= unique] identification numbers before going through the questionnaires to capture demographic information as well as animals/fowls kept at home” [This is repetition, see the above sub-title on socio-demographic characteristics]

 2 ^nd^ sentence

 “…whole blood was collected aseptically following vein puncture in the cubital fossa into sterile [insert=4.0 ml] plasma tubes (BD, Franklin Lakes, NJ, USA)…”

*CATT test and microscopy*

 Screening was performed using CATT/ *T. b. gambiense* in accordance to [= with] the manufacturer’s (ITM, Antwerp, Belgium) manual by diluting blood 1:2 in CATT buffer (ITM, Antwerp, Belgium).

**Comment:** By using CATT/ *T. b. gambiense* test alone, the authors seem to have overlooked the possibility of *T. b. rhodesiense* infections in the study area, even though past history of participants living in other regions was recorded. Yet they clearly state that “the so called frontier that divides the two pathogenic *brucei* subspecies separated by a distance of about 160km in northern Uganda seems to be only imaginary” This is supported by recent findings by Opiro et al., 2017.

 - Does CATT/ *T. b. gambiense* kit detect *T. b. gambiense* only?

 Based on the findings of LAMP assay used in this study, the authors have raised doubts on its sensitivity to detect *T. b. gambiense* and *T. b. gambiense.*

 2 ^nd^ sentence

 “…corresponding amount of the reagent and rotated onto [= on] a flat orbital rotator for 5 minutes at 60 rpm”

 3 ^rd^ sentence, on page 4

 “Both positive and negative controls were set along [=alongside] test samples and results read as positive, if there were visible agglutinations with the naked eye” *rephrase the statement to make it succinct, i.e if agglutinations were observed.*

 Last sentence of 1 ^st^ paragraph;

 “Titre was read as the highest dilution where visible agglutination [Delete the word ‘visible’] was observed”

 Next paragraph, 1 ^st^ sentence; page 4

 Wet preparations [of what?] were made, mounted with cover slips 24 × 32 mm and examined using Olympus CX21 microscope under x20 [= 20×] and x40 [= 40×] magnifications and the results recorded.

 2 ^nd^ sentence (page 4)

 “…for laboratory examinations under oil immersion (x100)” rephrase as “…for laboratory examinations under oil immersion using 100x objective”

*Sample preparations & detection of repetitive insertion mobile element (RIME) using loop mediated isothermal amplification (LAMP)*

 1 ^st^ sentence, page 4

“Homogenized whole blood from plasma tubes were sucked in heparinized capillary tubes sealed at one end…”

- How was homogenization of blood conducted?
- This blood collected in heparinized plasma tubes does not clot, why was homogenization necessary prior to preparation of Buffy coat?

 

**Data management and analysis (Page 4)**

 1 ^st^ sentence: “…converted to comma delimitated (.csv) file…” replace the word ‘delimitated’ with the correct one as ‘delimited’

 5 ^th^ sentence: “Bivariate logistic regression was performed to determined association between…” Replace ‘determined’ with ‘determine’

 Last sentence: “Any variable with P-value ≤0.05 was taken as a significant predictor of HAT presence” Replace ‘taken’ with ‘considered’

**Ethical clearance (Page 4)**

 3 ^rd^ sentence: “The school management (Head teacher) was briefed about the study and the pupils were briefed about the study by the investigators” The word ‘briefed’ is repeated two times, replace one with another appropriate word, e.g. ‘informed’

 6 ^th^ sentence: “…had first assented to participate in the study were excluded from the study…” delete “from the study” to avoid repetition of “the study”

**Results (Page 4)**

 3 ^rd^ paragraph, 1 ^st^ sentence: “School enrolment is at 50–50 for girls and boys although those involved in the study where mainly in the age group 13–18 years” Replace ‘where’ with ‘were’.

 3 ^rd^ paragraph, 2 ^nd^ sentence: “Fowls/animals that are mostly kept at home in the district are chicken, goats, and dogs with pigs and cattle are steadily on the increase over the years” Replace “with — pigs and cattle…” with “while — pigs and cattle...”

 4 ^th^ paragraph: “With the use of GPS; villages, schools, sub-counties, district headquarter, hospital/health centres and salient features such as district and sub-county boundaries, road networks and streams/rivers are located on the district map for ease of follow ups especially the CATT positive cases even by someone who has not been involved in the study (Table 3; Figure 1)” This long sentence does not provide relevant information because the exact GPS coordinates are not given at all — even under **Figure 1**. Again **Table 3** is not about GPS coordinates, but predictors of being CATT positive — thus remove citation of **Table 3** in this sentence.

**Table 1 (Page 5):**

 State the significance of titres at ½, ¼,⅛, 1/16, etc. Briefly, highlight the key message presented in the Table. The GPS coordinates could be provided in this Table that summarizes all surveyed schools.

**Table 2 (Page 5):**

 The words kept “at home” are repeated nine (9) times. Remove these words from the Table and retain under the description.

 Organize this Table by adding; specific sampling sites, GPS coordinates, number of participants from specific sites, the livestock species kept at home, etc.

**Table 3 (Page 6):**

 The basis for selection of some domestic livestock for inclusion in this study is not highlighted. What’s the key message in this Table 3? Why is the row containing ‘dogs’ highlighted in bold, unlike all others in the Table?

**Figure 2 (Page 7):** “(a) Prepared tests samples…” replace “tests” with “test”

**Discussion (Page 7)**

 3 ^rd^ sentence: “…the difference in the sexes was not statistically significance...” Replace ‘significance’ with ‘significant’

 4 ^th^ sentence: “…sexes in terms of infectivity…” Replace ‘infectivity’ with ‘infection’

 3 ^rd^ paragraph, page 8: “…75% of those who were found to be CATT positive where parasitologically positive with careful wet preparations and thick blood smear microscopy” Replace ‘where — parasitologically positive’ with ‘were — parasitologically positive’

 3 ^rd^ paragraph, last sentence page 8: “Humans, like animals, are believed to possess trypanotolerance that protect them from the disease” This statement is misleading because it generally implies that animals [not clear if domestic or wild] are trypanotolerant. It is better to name specific animals that are known to be trypanotolerant; only some breeds of cattle are trypanotolerant e.g. some West African Taurine cattle, whereas many others are susceptible.

 4 ^th^ paragraph, page 8: “Cut-off titres at some points were used to define disease cases; 1:4 required parasitological confirmation whereas 1:16 was regarded as indication of infection that required treatment even without parasites being seen” This information provides the answer to my earlier question in Table 1 (page 5) on the significance of different titres. Move this to come under Materials and Methods, subsection on “ *CATT test and microscopy” *(page 3).

 5 ^th^ paragraph, 1 ^st^ sentence, pg 8: “Though it has now been proven that animals play a yet unclear role in the maintenance of *T. b. gambiense* even when they have been eliminated from the human population, explaining in parts the reason behind re-emergence of HAT” Check again the flow of information in this sentence, re-write to make it clear.

 5 ^th^ paragraph, 1 ^st^ sentence, pg 8: “In our study however, we did not sample the animals but instead tried looking at possible associations of those pupils who were CATT positive and the domesticated animals. After analysis for possible associations we found that those who had dogs were more than 3 times at increased risk of being CATT positive” This statement is too speculative and oversimplified since the animals were not screened for trypanosome infection by PCR and gene sequencing. This is very important experiment for determining livestock reservoirs of the HAT. It will be impossible to pinpoint or even propose, without bias, the contributions of dogs in the disease epidemiology if the complex interactions between humans with other domestic, and wild animals are not well understood. In my opinion, the authors should avoid generalizations on the potential animal reservoirs of HAT due to lack of conclusive data.

**Study limitations (Page 8)**

 “This was a one off survey and therefore we cannot account for information on pupils who refuse to participate or were absent from school nor children who have dropout of schools” Rephrase the sentence, replace ‘refuse’ with ‘did not assent’, and also replace ‘dropout’ with ‘dropped out’

**Recommendations/conclusion (Page 8)**

 2 ^nd^ sentence: “There is need to for active screening of populations…” Delete the word “to”

I confirm that I have read this submission and believe that I have an appropriate level of expertise to confirm that it is of an acceptable scientific standard.
